# Probiotics for immunomodulation in prevention against respiratory viral infections with special emphasis on COVID-19

**DOI:** 10.3934/microbiol.2022024

**Published:** 2022-09-15

**Authors:** T. Amrouche, M. L. Chikindas

**Affiliations:** 1 Food safety and quality laboratory, Faculty of Biological Sciences and Agronomy,M. Mammeri University, Tizi-Wezzu, 15 000 Algeria; 2 Department of Food Science, Rutgers, The State University of New Jersey, 65 Dudley Road, New Brunswick, NJ 08901-8520, USA

**Keywords:** probiotics, immunomodulation, SARS-CoV-2, COVID-19, gut, lung

## Abstract

COVID-19 pandemic is currently causing high mortality and economic crisis, and several drugs-based therapeutic strategies and vaccines are unfortunately used with little efficiency. Therefore, here is an urgent need to provide additives therapies that prevent or improve symptoms in populations infected by SARS-CoV-2 variants. This review aimed to examine relevant scientific information related to SARS-CoV-2 and host antiviral immunity, as well the possible role of probiotics in gut-lung cross talk pathways to promote lung immune response to COVID-19 infection. We searched online databases such as PubMed, Embase, Chinese databases, and selected articles and studies with relevant data reported on COVID-19 and other respiratory viral infections. Recent research highlighted potential immunomodulatory activities of probiotics assessed in animal models and clinical trials. However, the role of probiotics and gut microbiome in COVID-19 management, and approaches with significant understanding in molecular mechanism of probiotic action remain poorly investigated. Clinical investigations as well as animal model studies published have demonstrated that probiotics such as *Lactobacillus rhamnosus* and *Bifidumbacterium lactis* HN019, may influence positively not only microbiota balance but also antiviral immunity by improving both innate and adaptive responses and controlling inflammatory reaction in respiratory viral infection. Given the immunological interactions in gut-lung axis and the crucial role of probiotics in modulating immune responses by promoting dendritic cells (DCs) to regulate T cell responses, we hypothesized that application of probiotics may be successful in prevention or treatment of both intestinal disorders and airway diseases in patients with COVID-19.

## 1. Introduction

Global health crisis causing loss of life and severe human suffering is raised by a novel coronavirus outbreak (COVID-19) due to SARS-CoV-2 variants Alpha, Beta, Gamma and Delta demonstrating variable phenotypic characteristics (transmissibility, disease severity, risk of reinfection, and vaccine performance) and resulting in over 559 million reported cases and 6.3 million deaths globally since the start of the pandemic [1]. COVID-19 outbreak causes not only high mortality but also has negative impact on social and economic fronts. This pandemicnamed as severe acute respiratory syndrome coronavirus 2 (SARS-CoV-2) has been detected for the first time in China and then rapidly has spread worldwide [2]. COVID-19 infection is frequently asymptomatic but the virus is virulent and transmitted quickly from individual to individual [3].

The infection remains exceptionally severe or fatal in vulnerable population (elderly, pregnant, comorbidities) [4],[5] and prevention of viral transmission is generally focused on basic measures including social distancing, hand washing, lockdown and quarantine. Unfortunately, there is no efficient therapeutics used in clinical setting to resolve the infection caused by SARS-CoV-2. T tentative therapies useful for COVID-19 patients including immunomodulatory agents, antiviral therapy, and vaccines reduce the severity of illness for high-risk patients. Currently, intensive research and clinical trials are supported to accelerate the development and production of vaccines and therapeutics, as well as diagnostic tools for COVID-19.

As no effective treatment is available as yet, people are looking for an alternative letting protective immunity enhancement by reinforcing own immune function against COVID-19. The best way to strength naturally own immunity is consumption of either food supporting gut microbiota like prebiotics, known as plant fiber stimulating selectively beneficial intestinal bacteria, or probiotics. WHO and FAO defined probiotics as live microorganisms when administered in adequate amounts confer a health benefit on the host assuming that they are alive and abundant once ingested. According to Zendeboodi et al. [6], probiotics may be classified as true probiotic referring to viable and active probiotic cell, pseudo-probiotic considered as viable and inactive cell (vegetative or spore) and ghost probiotic referring to dead/nonviable cell (intact or ruptured cells). One of the most promising health effects of probiotics in human is modulation of local and systemic immune responses [7]. Probiotics with immunomodulatory effects are referred as immunobiotics. Nowadays, probiotics as complements or foods containing probiotics are popular due to their benefits on health and are gaining increased attention in consumers, researchers and medicinal as well as food industry [8].

On the other hand, recent research has reported a cross talk between gut and lung system displaying interactions that induce immunological processes in host infection [9]. Since a close relationship between both organs is currently recognized, an effective probiotic-based approach may have an important role in dropping viral infection such as COVID-19. In this regard, recent findings suggest that probiotics may influence positively host antiviral immunity, but mechanisms by which probiotics may be effective to promote antiviral immunity are not fully known. Up to now, little is known about the role of probiotics and microbiome associated with gut immunity in COVID-19 patients. This review is an attempt to critically analyze perspectives for implementation of probiotics as immuno-modulators aimed at prevention of respiratory viral infections. Our review was carried out using online databases such as PubMed, Embase, Chinese databases and Google Scholar. We examined 200 articles and studies and selected 80 of them for review based on following criteria: randomized, blinded studies, unbiased articles and relevant in vivo studies. Institutional reports were used to provide some updated data on COVID-19.

## 2. Immunity and SARS-CoV-2

SARS-CoV-2 is a newly betacoronavirus identified from patients with pneumonia in China. This emergent coronavirus was found highly virulent as it was capable of infecting and replicating robustly in human lung [10]. This coronavirus was described as an enveloped non-segmented positive-sense RNA virus, which can be transmitted quickly by person-to-person contact, airborne [11], or oro-fecal way [12]. SARS-CoV-2 genome (RNA) encodes 16 non-structure proteins, 4 essential structural proteins including spike glycoprotein (S), envelope protein (E), matrix protein (M), nucleocapsid protein (N), and accessory proteins. S glycoprotein allows virus entry in host by binding cell receptors [3]. Clinical symptoms of COVID-19 were described as fever, cough, breath shortness, and lung invasive lesions. Incubation period of SARS-CoV-2 was 3 to 7 days and infection of respiratory tract can cause pneumonia with acute respiratory distress syndrome and organ failure [13].

Immune system induces both innate and adaptive immune responses to strongly faith infectious agents like pathogenic virus. Innate immunity cells act in first line against pathogens and then instruct specific adaptive immune responses. It has been reported that innate immune system plays a key role in antiviral response via virus replication suppression, virus clearance and adaptive immune response stimulation [14].

Studies reported on human airway disease indicated that immune response is essential to viral clearance. Zhou et al. [15] explored disease progression in a cohort of patients infected with novel coronavirus and suggested that immune response may be critical in controlling disease progression at the early stage of viral infection. However, inappropriate immune responses may result in immunopathology and lung impairment. Undeniably, unsuitable antiviral immunity may lead to massive inflammatory responses and persistence of inflammation in lungs of patients with viral infection. Furthermore, immunosenescence in elderly results in immune proficiency deterioration characterized by systemic and chronic inflammation as well as innate/adaptive immune imbalance especially in elderly patients with cardiovascular diseases [16].

SARS-CoV-2 structural proteins like spike protein interacts with host cells key receptor ACE2 and target directly immune system components leading to immune vigilance deficiency [17]. Interaction between viral surface proteins and host cell receptor ACE2 should be prevented to escape infection with SARS-CoV-2, thereby improving lung protection against injury or failure [18]. Baruah and Bose [19] have recently revealed significant cytotoxic T lymphocyte and B cell epitopes that are uniquely present on SARS-CoV-2 surface glycoprotein and potentially engaged in antiviral immunity when viral particles are detected by host cells.

Recent research reported that COVID-19 may results in lung injury accompanied by uncontrolled production of pro-inflammatory mediators engendering cytokine storm syndrome [20] shown by higher plasma levels of granulocyte-colony stimulating factor, interferon gamma-induced protein 10, monocyte chemoattractant protein 1, macrophage inflammatory protein 1α and tumour necrosis factor α [13]. COVID-19 severity was linked to cytokine storm causing acute respiratory distress syndrome especially in patients with comorbidities (diabetes, hypertension, cardiovascular disease, etc.) [21].

Viruses are detected in host by sensing pathogen-associated molecular patterns (PAMPs) via transmembrane and cytosolic receptors expressed in respiratory epithelial cells and immune cells, referred as pattern recognition receptors (PRRs). Among transmembrane receptors, toll-like receptors (TLRs) play a crucial role in antiviral immunity as they detect both live viruses and viral particles (nucleic acid and envelop proteins) while cytosolic innate receptors are less active enabling only recognition of live viruses [22],[23]. Respiratory epithelial cells and infiltrating leukocytes produce large amounts of antiviral molecules, such as type I IFN involved in a complex cross-regulatory talk between IFNs and neutrophils initiating appropriate antiviral immune responses with minimum tissue damage [24].

Antiviral response in respiratory system is initiated by innate immune inducing expression of cytokines and chemokines letting activation and differentiation of dendritic cells (DC), macrophages, neutrophils and NK cells, and other elements of innate immune system that are engaged in pathogen replication suppression and its elimination [20],[25],[26]. Thereby, cell innate immunity and professional antigen presenting cells are activated by pathogen sensing and induce a potent initial inflammatory response that stimulate specific adaptive immune reactions via B and T lymphocytes [27].

In order to prevent massive production of cytokines involved in proinflammatory cytokine storm immunoytherapy is currently applied using immunomodulatory agents that alleviate hyperinflammation symptoms in COVID-19 patients with respiratory failure [28]. Zhu et al. [11] evaluated the clinical value of immune-inflammatory parameters to assess the severity of infection in 127 hospitalized patients infected by SARS-CoV-2 and demonstrated that IL-6 played a key role in the severity of COVID-19 disease. A specific monoclonal antibody, namely Tocilizumab, directed against IL-6 has recently been shown to improve clinical outcome immediately [12], and recommended by National Health Commission of China [29] for treatment of patients with lung lesions and high IL-6 level.

## 3. Probiotics for the immune system modulation: immunobiotics

Among preventive and therapeutic options for severe COVID-19 discussed or approved till now, immunotherapy based on use of probiotics as immunomodulatory agents remains neglected despite their potential to modulate host immunity. Currently, immunobiotics are gaining more attention considering their potential to confer protection against viral infections by modulating innate and adaptive antiviral immunity [30]. In addition, specific microbial strains resident in gut ecosystem called next-generation probiotics were shown to have health beneficial effects, and considered as food/nutraceutical supplements and biotherapeutic products [31]. Novel microbial strains are being tested for their potential use in prevention and treatment of many diseases such as antiviral therapies. However, clinical studies are still scarce and approval from regulatory agencies is rare [32]. For example, *Faecalibacterium prausnitzii*, *Akkermansia muciniphila* and *Eubacterium hallii* have been identified as next generation probiotics with potential for the prevention and treatment of dysbiosis-associated diseases [33]. Zhang et al. [34] suggested that gut microbiota-derived synbiotic (Bifidobacteria strains and prebiotics) stimulated antibody formation, reduced nasopharyngeal viral load, reduced pro-inflammatory markers, and restored gut dysbiosis in COVID-19 patients.

Both viable and non-viable immunobiotics (cytoplasm, cell wall and exopolysaccharides) were reported to influence positively immune function [35],[36] and to decline the severity of infections in gastrointestinal [37] as well in respiratory tracts [38],[39]. Indeed, lactic acid bacteria were shown to have potential to improve human and animal health via modulation of mucosal and systemic immune responses [40]. Moreover, treatment with lactic acid bacteria probiotic strains has been associated with gut microbiome improvement and allergy and gastrointestinal disorders reduction [41].

Probiotics are usually recommended to general consumer to improve health, but immunobiotics such as some lactobacilli [42] and bifidobacteria [37] could be particularly useful to enhance immune responses in infants or elderly. Additionally, such probiotics could be helpful to people with dysbiosis (microbiota imbalance) caused by antibiotics intake, stress, exposure to toxins, disease, excessive exercise, etc. [12]. A randomized and controlled clinical trial was conducted on 37 patients with cystic fibrosis to determine the effect of immunobiotics on gut microbiota and immunity, besides intestinal function enhancement, significant reduction of pulmonary exacerbation was observed among immunobiotics patient group in comparison with placebo group [43]. Microbial patterns manipulation through the use of probiotics and dietary fibers consumption may have anti-inflammatory effects in COVID-19 infection [44].

## 4. Immunobiotics and viral respiratory infections

Current immunology challenge is to find alternatives approaches for immune system modulation in individuals suffering from severe infections such as airways infections, and autoimmune diseases. Several research works have demonstrated that immunobiotics are a potential alternative to improve outcomes of viral infections [45]. However, studies performed on immunobiotics in clinical conditions are not easy to extrapolate due to heterogeneity in patient population and immunobiotics, and combination of products used [46]. Although immunobiotics seems to have modest impact on prevention of human viral respiratory infection [47], there is growing evidence that certain immunobiotics may be effective in modulating the gastrointestinal tract-mediated antiviral innate immunity. It has been reported that treatment with Lactobacillus strains reduced severity of respiratory tract infection of viral origin [48].

Studies carried out on animal models with virus challenge to evaluate immunobiotics administration effects on host immune responses [38],[49],[30] demonstrated that treated groups display impacts on baseline state of innate immunity against viral infection. In this regard, Eguchi et al., [50] demonstrated that mice orally administrated with *Lactobacillus gasseri* SBT2055 exhibited significant reduction of infection in lung of mice challenged with respiratory syncytial virus. Likewise, mice immunized with *Lactobacillus plantarum* expressing antigen from gastroenteritis coronavirus were shown to stipulate both humoral and cell-mediated responses [51]. Several randomized controlled trials performed on immunobiotics used for volunteers and hospitalized patients with respiratory infection have been published (Table 1).

**Table 1.** Antiviral effects of different immunobiotics used in clinical studies.

Immunobiotics	Experimental Challenge	Study population	Study design	Antiviral outcomes	Ref
*Lactobacillusrhamnosus GG*	COVID-19 infection	1132 subjects tested positive for COVID-19	Double-blinded, randomised, placebo-controlled trial. Daily oral administration of LGG or placebo 28 days	Reduction of secondary infection and moderation of immunity in patients	[52]
*Bifidobacterium, Lactobacillus, Enterococcus and Bacillus*	COVID-19 infection	311 patientsAge ≥ 18 years	Single-center retrospective analysis 1.5 g (tablet) 28 days	Reduction of severity of disease and associated with beneficial changes in gut microbiome composition	[53]
*Lactiplantibacillus plantarum* KABP022, KABP023, KAPB033, *Pediococcus acidilactici* KABP021	COVID-19 infection	300 adult Covid19 outpatients Media age: 37 years	Single-center, quadruple-blinded, randomized trial 2 × 10^9^ CFU/day or placebo 30 days	Reduction of nasopharyngeal viral load, lung infiltrates Increase of specific IgM and IgG level	[54]
*Bifidobacterium longum Lactobacillus bulgaricus Streptococcus thermophilus*	COVID-19 infection	156 patients	Randomized controlled trial 4 x 0.5 × 10^6^ CFU, 3 times a day	Significant decrease of time to achieving a negative nucleic acid test and inflammation indexes	[55]
Bifidobacteria strains, galactooligosaccharides xylooligosaccharide, resistant dextrin	COVID-19 infection	55 patients Age ≥ 18 years	Open-label, proof-of-concept study 10 x 10^11^ CFU/day 28 days	Significant reduction in pro-inflammatory markers (IL-6, MCP-1, M-CSF, TNF-α and IL-1RA) compared with controls	[56]
Heat-killed *Pediococcus acidilactici K15*	Viral respiratory tract infections	172 children Age 3–6 years	Randomized, double-blind, placebo-controlled 5 × 10^10^ bacteria4 months	Duration of a fever significantly decreased sIgA level significantly higher	[57]
*Lactiplantibacillus plantarum HEAL9 and Lacticaseibacillus paracasei 8700:2*	Common colds	448 Age 18–70 years	Double-blind study 10^9^ CFU/day 12 weeks	Reduction of symptom severity IFN-γ production enhancement	[58]
*Bacillus spores (LiveSpo Navax)*	Acute respiratory tract infections	46 children	Nasal-spraying of spores 5 × 10^9^ spores 6 days	Symptoms improvement Reduction of load and level of pro-inflammatory cytokines	[59]
*Bifidobacterium animalis* subsp. lactis Bl-04 (1)	Respiratory and gastro intestinal infections	241 males; age 35 years and 224 females; age 36 years	Randomized double-blind placebo-controlled trial. 2.0 × 10^9^ CFU/day 3–4 months	Significant decrease in respiratory illness episode compared to placebo.	[60]
*Lactobacillus plantarum* HEAL9 and *Lactobacillus paracasei* 8700:2	Common cold	131 childrenAge 1–6 years	Randomized, double blind placebo-controlled trial. 10^9^ CFU/day 3 months	Significant decrease of symptom severity	[61]
*Loigolactobacillus coryniformis K8 CECT 5711*	Immune response generated by the COVID-19 mRNA vaccine	200 subjects > 60 years	Randomized, placebo-controlled, double-blind trial 3 months	Levels of IgG were significantly higher	[62]
*Lactobacillus rhamnosus* HN001	Acute respiratory infections	398 children Age 1–5 years	Double-blind placebo-controlled trial. 10^10^ CFU/day 3 months	High level of Lactobacillus Significant increase of sIgA levels in treated group	[63]
*Saccharomyces cerevisiae*	Cold and flu-like symptoms	116 subjects 57% femalesAge 18–94 years	Randomized double-blind placebo-controlled trial 500 mg (dried)/day 12-week	Significant decrease in incidence, no significant reduction in duration, no impact on severity of illness	[64]

Tang et al. [52] demonstrated that daily oral administration (for 28 days) of the probiotic *Lactobacillus rhamnosus* GG versus placebo on COVID-19 infection status and gut microbiome in 1132 COVID-19 patients Ddecreased disease severity (fever, chills, headache, muscle aches, diarrhoea, etc.) associated with beneficial changes in gut microbiome composition. Li et al. [53] collected in a retrospective single-center study data of 311 COVID-19 patients in Wuhan (China), and analyzed and compared epidemiological, clinical and medication characteristics of patients with versus without probiotics (*Lactobacillus rhamnosus* GG). They found that probiotics could not reduce the increased IL-6 levels but moderate the immunity and decreased the incidence of secondary infection in COVID-19 patients.

Recently, Gutiérrez-Castrellón et al. [54] investigated the impact of probiotics on symptomatic and viral clearance in COVID19 outpatients via a randomized, quadruple-blinded, placebo-controlled trial. Their findings suggested that probiotic supplementation (*Lactiplantibacillus plantarum* and *Pediococcus acidilactici*) reduced both digestive and non-digestive symptoms, compared to placebo. They reported that probiotics primarily interact with immune system rather than changing colonic microbiota composition in COVID19 patients.

Shin et al. [65] reported that 112 individuals with abnormal bowel movement symptoms enrolled in a randomized, double-blinded, placebo-controlled trial using a probiotic preparation containing *Lactobacillus johnsonii* IDCC 9203, *Lactobacillus plantarum* IDCC 3501 and *Bifdobacterium lactis* IDCC 4301 did not exhibit any alteration in overall gut microbial composition after treatment. Their data demonstrated that probiotic administration leads to decrease in symptoms and increase in gut microbial abundance of *Lactobacillus johnsonii* and *Bifdobacterium lactis*. Probiotic intervention without triggering any severe adverse effects suggests that probiotic administration in patients may be both safe and effective. Recently a randomized controlled study performed on effectiveness and safety of multi-strain probiotic preparation (mixture of Lactobacillus, Bifidobacterium, and *Streptococcus thermophilus* strains) in patients with diarrhea-predominant irritable bowel syndrome showed a significant improvement in symptoms in patients and demonstrated that probiotic preparation was well tolerated and safe [66].

However, even though probiotic was considered as generally recognized as safe (GRAS), they may have side effects including systemic infections, immune stimulation, metabolism alteration [67]. Also, the safety of probiotics used should be considered since probiotic bacteria were found to have the potential to transfer antibiotic resistance genes to commensal or pathogenic bacteria present in gut ecosystem. For example, transmission of antibiotic resistance genes to strains between Lactobacillus strains (*L. plantarum*, *L. reuteri*) and other lactic acid bacteria occurs via pAMβ plasmid [68]. In few clinical trials probiotic use was found to have side effects such as promoting inflammatory cascade rather than suppressing it [69].

Furthermore, probiotics are known for their potential to produce bacteriocins described as antimicrobial peptides controlling clinically relevant susceptible and drug-resistant bacteria. Bacteriocins have been studied in animal models and were found to have many positive effects in the host such as modification of immunogenic response, alteration of inflammatory response, and reduction of biochemical and histopathological parameters related with infection. But no side effects or toxicity assays were described and data on toxicity and biosafety studies of bacteriocins are crucial to make progress into clinical trials [70].

A randomized, double-blind, parallel and placebo-controlled study carried out by Anaya-Loyola [71] assessed the effect of *Bacillus coagulans* GBI-30 against both upper respiratory and gastrointestinal infections in children. It was shown that daily administration of probiotic (1 × 10^9^ CFU) or placebo for three months significantly decreased the duration of infection-associated symptoms and allowed modulation of serum TNFα, CD163, G-CSF, ICAM-1, IL-6, IL-8, MCP-2, RAGE, uPAR, and PF4.

Rodriguez et al. [72] conducted a single-center, randomized, double-blind, placebo-controlled study in 72 volunteers who received a synergistic combination of yeast-based ingredients: glucan complex and a consortium of heat-treated probiotic *Saccharomyces cerevisiae* after vaccination against influenza or COVID-19. They demonstrated increased levels of CD4+, CD3+ and CD8+T lymphocytes in COVID-19 cohort, as well as higher levels of IgG and IgM in volunteer's serum.

## 5. Immunobiotics intervention in gut-lung pathways

### 5.1. Gut–lung axis

**Figure 1.**
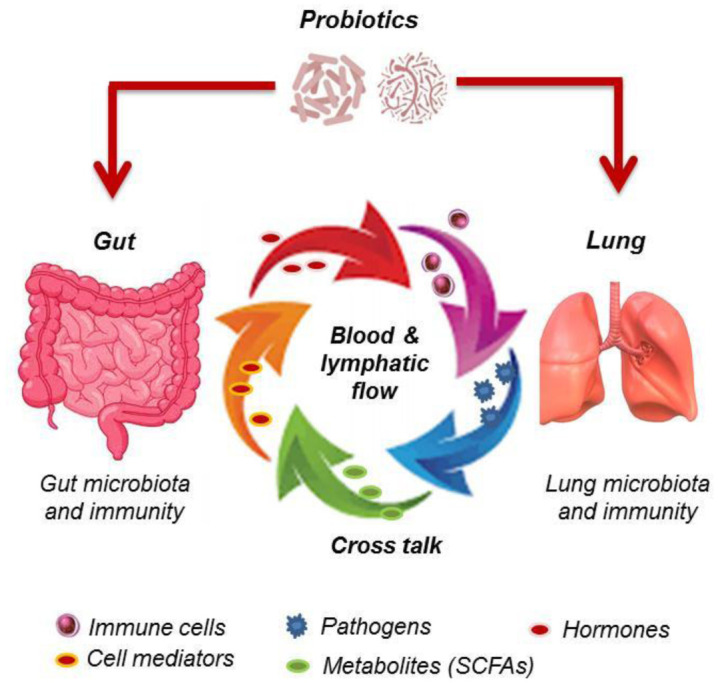
Bidirectional talk-cross between human gut and lung. Gut and lung are connected via blood circulation and lymphatic flow driving elements such as immune cells and mediators, and friendly or harmful microbes (virus or bacteria) as well as components or metabolites that naturally maintain microbiota and immune system of both organs permanently interactive. These interactions may be stimulated by probiotics influencing local and distant microbiota and immune system in host.

Scientific consensus established that respiratory system, like gastrointestinal system, needs helpful and balanced microbiota to preserve health. Some research dedicated to the relationship between gastrointestinal tract and lung system in terms of pathogenesis and health demonstrated that intestinal microbiota activity as well as immunobiotics intervention impacts lung immunity through an active and complex bidirectional cross-talk between gut and lung, referred as gut-lung axis [73],[74]. Simplified interactions occurring in human gut-lung axis are schematically presented in Figure 1.

Probiotic secondary metabolites, known as postbiotics, exhibit potential beneficial effects in humans like prevention or treatment of inflammatory diseases, diabetes, neurological disorders, etc. Among compounds described vitamins, amino acids, or antimicrobials are gaining great interest [75]. Anwar et al. [76] revealed antiviral effects of probiotic metabolites on COVID-19 via a molecular dynamics study using metabolic product of *Lactobacillus plantarum* that blocks the entry by binding SARS-CoV-2. They demonstrated the strengthen stability of the complexes of plantaricin w and SARS-CoV-2 RdRp enzyme, residual binding protein on spike proteins, and human ACE2 receptor.

The connection between gut and lung occurs via lymphatic system and blood circulation carrying immune cell mediators like cytokines, microbial fragments or products (peptidoglycan, endotoxins, proteins, short-chain fatty acids (SCFAs)), and hormones [9]. However, interactivity between lung diseases and gut microbiota is not clearly elucidated. Respiratory virus infection was reported to cause perturbations in gut microbiota [74]. It has been reported that microorganisms and/or their fragments may reach lungs via translocation and blood circulation from gut to lung, vice versa [77].

The intestine is highly colonized by microbiota that plays an important role in modulating host-pathogen interactions. Microbial population inhabiting gut holds more than two thousand species, and has been estimated to exceed 10^14^ [78]. Lung bacterial biomass is relatively fewer when compared to gut microbiota; because of low nutrient resource and physiological conditions (oxygen tension, pH, temperature, etc.) the lung does not favor microbial growth [79]. According to Sze et al. [80], lung tissues display some 10–100 bacteria per 1000 human cells. Among phyla of bacteria present in human gastrointestinal tract Firmicutes (*Lactobacillus spp*., *Bacillus spp*., and *Clostridium spp*.) and Bacteroidetes (*Bacteroides*) are being prevalent, and Proteobacteria (*Escherichia*) and Actinobacteria (*Bifidobacterium*) less abundant [81] whereas Bacteroidetes, Firmicutes, and Proteobacteria predominant among lung microbiota [79].

Growing evidence suggests that alterations in lung and/or gut microbiota cause dysbiosis increasing inﬂammation and acute lung injury accompanied by exacerbations of symptoms in affected host. Burke et al. [82] by assessing the effect of cystic fibrosis (affecting pulmonary and intestinal) on gut microbiota of patient group versus healthy controls demonstrated significant alteration of gut microbiota shown by declined microbial diversity and Bacteroidetes, and increased Firmicutes in patients with cystic fibrosis. Growing research proves that modifications in gut microbiota are linked to alterations in both immune responses and disease progression in lungs [83]. As ACE2 is present in intestinal cells, a possible cross-talk between lung and gut occurs in COVID-19 patients. Diarrhea associated with viral nucleic acids detection in fecal samples of COVID-19 patients could explain partially gut-lung interactions [12]. Furthermore, a systemic positive effect was found to be induced by gut microbiota via production of short chain fatty acids suppressing lung inflammation [73].

### 5.2. Immunological cross talk

Connections in gut-lung axis occur by an immunological process induced by microbiota, immunobiotics and pathogens (virus). The precise mechanisms underlying immunoregulatory activities of immunobiotics in antiviral immunity are not yet fully elucidated, however antiviral immunity against common respiratory viruses, including influenza, rhinovirus and respiratory syncytial virus has been documented [84]. Immunobiotics are postulated to have unique roles in modulating cross-talk between commensal bacteria and mucosal immune system [85]. Given the lack of data related to immunobiotics-based application in COVID-19 infection, we assess evidence from previous studies dedicated to immunobiotics-based immunotherapy relevant for viral diseases with special emphasis on respiratory infections. Immunobiotics like helpful gut bacteria may have an important role in supporting an appropriate immune response to respiratory viral infection. They may prevent alteration of distribution DCs as well as reduction of CD8(+) and CD4(+) T cells activation in lung viral infection [86].

Immunobiotics were reported to stimulate immune system via TLRs that are most closely correlated to adaptive immune reactions and play a crucial role in antiviral immunity. According to Kitazawa and Villena [24] the immunobiotic strain *Lactobacillus rhamnosus* CRL1505 acts by modulating of TLR3-mediated immune response against viral challenge in respiratory tract. Several immunobiotics strains have been screened for their immunomodulatory activities by Ho et al. [87], *Lactobacillus paracasei* BRAP01 and *Lactobacillus acidophilus* AD300 associated with higher value of IFN-γ/IL-10 were found to enhance the NK cytotoxicity in human blood. Immunobiotics such as *Lactobacillus rhamnosus* and *Bifidumbacterium lactis* HN019 that exhibit anti-inflammatory proprieties could be helpful for patients with COVID-19 [82]. Surface expression of dendritic cells-targeted spike antigen of coronavirus has been reported to stimulate effectively cellular, mucosal, and humoral immunity in mice [51].

In the context of severe viral infection, TLR activation for antiviral immunity mostly leads to induction of type I IFN, and then expression of this family of cytokines by all TLRs [88]. Bonjardim [89] stated that interferons as key cytokines involved in innate antiviral immune response are highly expressed by dendritic cells that recognize virus molecular patterns via TLRs. Hence, these cytokines induce subsequent expression of proinflammatory cytokines and costimulatory molecules resulting in activation of adaptive antiviral immunity. Given the potential immunomodulatory of immunobiotics it could be hypothesized that immunobiotics input may prevent uncontrolled production of proinflammatory mediators provoking cytokine storm in COVID-19 infection. Recently, it has been reported that lactic acid bacteria with immunobiotic effect modulate innate antiviral immune response in human intestinal cells via up-regulation of IFN-β and down-regulation of IL-6, IL-8, MCP-1, and IL-1β mRNA levels. Additionally, they amplified IFN-α and interleukin-10 (IL-10) and reduced tumor necrosis factor-α (TNF-α) and IL-1β protein/mRNA levels in human intestinal cells [36].

In animals *Lactobacillus plantarum* DK119 orally or intranasally administered to mice was found to enhance anti-influenza immunity by modulating innate immunity via dendritic cells and macrophage, and cytokine production pattern in mouse model [90]. Chiba et al. [42] explored immunological mechanisms involved in immunobiotic activity of *Lactobacillus rhamnosus* CRL1505 in mice challenged by respiratory syncytial virus infection. Their findings demonstrated that immunomodulatory effect in pulmonary microenvironment was induced by IFN-γ and IL-10 secretion, associated with activation of CD103(+), CD11b(high) dendritic cells, generation of CD3(+)CD4(+)IFN-γ(+) Th1 cells, and decrease of robust Th2 cell reactions.

**Figure 2.**
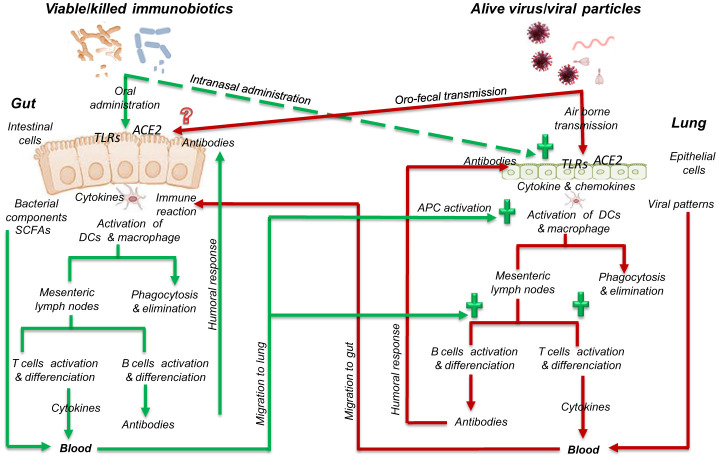
Simplified schema summarizing possible role of immunobiotics in immunogical interactions and pathways between gut mucosa and lung epithelium. APC, presenting cells; DC, dendritic cell; TLRs, toll-like receptors; ACE2, angiotensin-converting enzyme 2; SCFAs, short chain fatty acids. (+) Immunobiotics influence positively lung improving immune response (humoral and cell-mediated responses) and controlling inflammatory reactions that generate massive production of proinflammatory cytokines. Mechanisms by which alive virus or viral particles interact with gut cells and immune system are not yet understood.

Immunobiotics interact with strain specific TLRs that bind to bacterial surface-associated PAMPs (lipoprotein, peptidoglycans). Lactobacilli were shown to stimulate TLR1, TLR2 and TLR4, whereas Bifidobacteria activate TLR2 [25]. Immunobiotics intervention in immunological interactions and pathways between gut mucosa and lung epithelium is summarized in Figure 2.

Viable immunobiotics or fragments released by killed bacteria from host gut may reach lung by blood or lymphatic flow to promote its immune response similarly as done in gut [91]. Furthermore, immunobiotics were found to alter significantly the composition of *Lactobacillus spp*. and *Bifidobacterium spp*. as well as increasing total short chain fatty acids (SCFAs) and propionic acid contents in children gut [80]. SCFAs including acetate, propionate, and butyrate have been reported as metabolites regulating intestinal barrier function (via mucin secretion) and immunity through cellular receptor signaling. Moreover, SCFAs, particularly butyrate, have an important immunomodulatory potential that influences dendritic cell function, nuclear factors action, differentiation of regulatory T cells, interleukin-10 production, etc. [92].

Given the recognition of their immodulatory proprieties, immunobiotics could be considered to modulate immune responses in viral infection such as COVID-19 where loss of homeostatic equilibrium between Treg cells (IL-10) and Th17 cells (IL-17) was observed [82].

## 6. Conclusion

In this review we have highlighted an alternative approach founded on potential preventive or therapeutic application of certain probiotics in the context of viral respiratory infection. Considering unavailability of effective immunotherapy for COVID-19, bacteriotherapy based on immunobiotics as pharmabiotics or functional foods is a strategy that may directly or indirectly contribute to combat COVID-19 disease. We suggest that probiotics well documented for immunomodulatory and respiratory activities should be considered for intensive clinical trials to alleviate the duration and severity of COVID-19 disease. Use of immunobiotics may restore microbial dysbiosis, as well modulating antiviral immunity in patients with impaired lungs. The drug-independent strategy should be encouraged at least in combination with current therapeutics since anti-COVID-19 drugs are being tried with little efficiency to reduce disease severity and mortality.
